# Heparin-Induced Thrombocytopenia in a Patient With Pulmonary Embolism and Bilateral Deep Venous Thrombosis: A Case Report

**DOI:** 10.7759/cureus.83910

**Published:** 2025-05-11

**Authors:** Blessing T Ojinna, Arshia Ahmed, Lela Adeoshun, Zia H Shah

**Affiliations:** 1 Internal Medicine, Guthrie Lourdes Hospital, Binghamton, USA; 2 Global Health, Emory University Rollins School of Public Health, Atlanta, USA

**Keywords:** argatroban, deep venous thrombosis (dvt), heparin-induced thrombocytopenia (hit), hip antibody, pulmonary embolisim, serotonin release assay

## Abstract

The main non-bleeding complication arising from exposure to heparin is heparin-induced thrombocytopenia (HIT). Type I HIT is a non-immune-mediated mild decrease in platelet count, which mostly does not need treatment, and type II HIT is an immune-mediated, severe decrease in platelet count characterized by a significant risk of thrombotic complications requiring immediate treatment. HIT type II is a serious condition that can threaten life due to an immune and thrombotic response that continues to pose diagnostic and management challenges. Due to ineffective alternatives to heparin in certain typical and recurring situations, the disease burden remains unchanged in the U.S. HIT occurs in about 20,000 cases annually, representing one in every 1,500 hospital admissions. For patients diagnosed with pulmonary embolism (PE), heparin is often employed as an anticoagulant; however, the emergence of HIT in these cases complicates both the treatment plan and management approach. This case illustrates a patient who was diagnosed with a PE and received heparin therapy. Shortly after starting treatment, the patient experienced thrombocytopenia, a key indicator of HIT.

A 73-year-old male, while on admission, was noticed to have left upper extremity swelling and increased shortness of breath. A CT chest pulmonary angiogram revealed a positive for acute pulmonary artery embolus in the right lower lobar branch pulmonary artery. Vascular Laboratory (VL) left upper extremity duplex revealed acute occlusive deep venous thrombosis (DVT) of the left jugular, subclavian, axillary, and brachial veins and superficial vein thrombosis in basilic and cephalic veins. The patient received an IV heparin bolus and continued on heparin infusion. The platelet count on initiating the heparin drip was 145 K/μL. On hospital day 5, the patient's platelet count dropped to 98 K/μL.The 4Ts for HIT score calculated was 7 points, meaning high probability, and heparin-induced platelet (HIP) antibody was ordered, and heparin drip was discontinued. Argatroban infusion was started. The Hematologist evaluated the patient and stated that the clinical findings were consistent with HIT. The HIP antibody (screening test) resulted positive, and the patient's optical density (O.D) was elevated to 0.536. Later on, the patient was noted to have right upper extremity swelling, and VL duplex upper extremity right veins showed acute occlusive DVT involving the right subclavian vein, axillary vein, brachial vein, and internal jugular vein, and a drop in platelet count to 54 K/μL.

The unfractionated heparin (UFH) serotonin release assay, which is the confirmatory test for HIT, resulted positive result. Argatroban dose was increased to the maximum of the therapeutic range, aiming for a partial thromboplastin time of 70-80, as the patient continued to experience thromboses. This led to the recovery of platelets, discontinuation of Argatroban, and transition of the patient to Eliquis.

## Introduction

Heparin-induced thrombocytopenia (HIT), mostly type II is a serious and potentially life-threatening disorder that can arise after heparin administration. It is characterized by a reduction in platelet count and an increased risk of thrombosis. HIT usually manifests within five to 10 days after exposure to heparin, which can lead to life-threatening complications due to previous exposure, although it may occur sooner in individuals with a history of heparin use [1]. For patients diagnosed with pulmonary embolism (PE), heparin is often employed as an anticoagulant; however, the emergence of HIT in these cases complicates both the treatment plan and management approach. This case illustrates a patient who was diagnosed with a PE and received heparin therapy. Shortly after starting treatment, the patient experienced thrombocytopenia, a key indicator of HIT. Considering the associated risk of paradoxical thrombosis, it is vital to diagnose and promptly manage HIT in these patients to avert additional complications. Timely identification of this condition is critical, as it requires immediate discontinuation of heparin and the initiation of alternative anticoagulation therapy to prevent thrombotic incidents such as deep vein thrombosis, stroke, or recurrent PE [1,2].

The primary non-bleeding complication from exposure to heparin is HIT. HIT-II is a serious condition that can threaten life due to an immune and thrombotic response that continues to pose diagnostic and management challenges. Despite conditional alternatives like bivalirudin, argatroban, and fondaparinux to heparin in certain typical and recurring situations, they may not be the drug of choice, and this makes the disease burden remain unchanged in the U.S. HIT occurs in about 20,000 cases annually, representing one in every 1,500 hospital admissions. The highest risk for developing HIT is among patients who are undergoing cardiac surgery (0.6%) and those receiving dialysis for acute kidney injury (0.5%) [3-5].  In this case report, we are reporting a rare complication of heparin therapy, which is mostly used in patients admitted to the hospital for acute PE or deep venous thrombosis (DVT) treatment who are not candidates for mechanical thrombectomy and found to have ongoing thrombosis after discontinuation of heparin infusion.

## Case presentation

A 73-year-old Caucasian male, with a past medical history of chronic obstructive pulmonary disease, type 2 diabetes mellitus, benign prostatic hyperplasia, depression, peripheral artery disease, inflammatory bowel syndrome, obstructive sleep apnea, hyperlipidemia, hypertension, lung nodules, adrenal nodule, and diastolic congestive heart failure with preserved ejection fraction, arrived at the emergency department after receiving a call from his primary care provider’s office, informing him of an elevated white blood cell count and worsening kidney function. The patient's labs done by the primary care provider’s office showed leukocytosis of 18.49 K/μL, creatinine of 2.46 mg/dL, and glomerular filtration rate (GFR) of 27 mL/min, which was increased from his baseline creatinine of 1.00 mg/dL. The patient had been discharged for congestive heart failure exacerbation from the hospital three days prior to this presentation, during which he received subcutaneous heparin for DVT prophylaxis.

He stated that since discharge, he had persistent dyspnea on exertion when he walked more than five to 10 steps and a slight cough. In the emergency department, his vitals were stable. During respiratory examination, his lungs had coarse breath sounds at the bases bilaterally but were otherwise clear to auscultation. An earlier chest x-ray showed a developing pneumonia in the left lower lung. The patient was started on gentle IV hydration, given known acute kidney injury and signs of congestive heart failure exacerbation. On admission, he was started on subcutaneous heparin for deep venous thrombosis (DVT) prophylaxis. Labs on the day of admission are shown in Table 1.

**Table 1:** Labs on admission WBC: White Blood Cells; GFR: Glomerular Filtration Rate; BUN: Blood Urea Nitrogen

Related results	Reference range and units	Patient result
WBC	4.23-9.07 K/μL	19.81
Hemoglobin	13.7-17.5 g/dL	13.6
Hematocrit	40.1%-51.0%	40.9
Platelet count	163-337 k/Ul	152
Sodium	136-145mmol/L	131
Creatinine	0.67-1.17 mg/dL	2.76
GFR	>60 mL/min	24
BUN	8-23 mg/dL	69
Urine sodium	30-90 mmol/L	<20
Troponin	<0.04 ng/mL	0.03
Urine appearance	Clear	Turbid
Urine protein	Negative (mg/dL)	70
Urine hyaline cast	None seen/LPF	3-5

**Figure 1:**
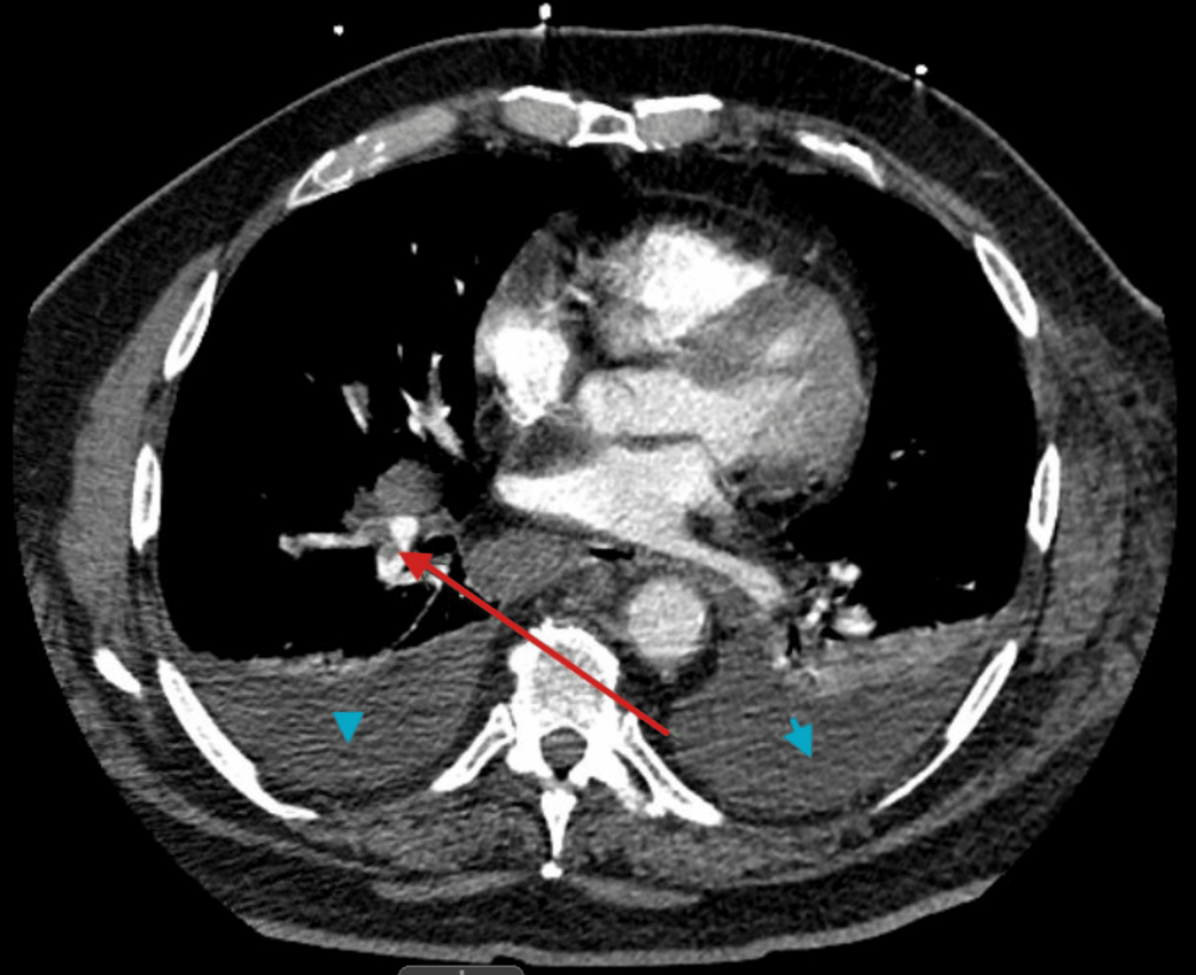
Computed tomography pulmonary angiogram axial image showing filling defect consistent with pulmonary embolism marked by red arrow and blue arrow heads showing bilateral pleural effusion.

On the third day of admission, the patient developed increased shortness of breath, required 2L of supplemental oxygen via nasal canula, and was noted to have left upper extremity swelling. A CT chest pulmonary angiography revealed an acute pulmonary artery embolus in the right lower lobar branch pulmonary arteries; however, no evidence of right ventricular strain. There were also noticeable moderate bilateral pleural effusions with adjacent consolidation, which was thought to be compressive atelectasis and a nonspecific mediastinal adenopathy. Figure 1 shows a CT chest pulmonary angiogram of pulmonary embolism and bilateral pleural effusions.

The VL left upper extremity duplex revealed acute occlusive deep venous thrombosis in multiple veins, including the left jugular, subclavian, axillary, and brachial. It also showed superficial vein thrombosis in the basilic and cephalic veins. The patient received a loading dose of IV heparin 7,000 units and heparin infusion at 18 units/kg/h with monitoring of partial thromboplastin time (PTT) and to adjust drip to DVT/PE protocol. The platelet count on initiating the heparin drip was 145 K/μL. Vascular surgery was consulted, and they recommended that the patient be scheduled for left upper extremity thrombolysis after improvement of kidney function. On admission day 5, the patient's platelets were noted to be 98 K/μL, which resulted in a diagnosis of thrombocytopenia, with HIT as the most likely cause. The 4T score for HIT was 7 points, signaling high probability. Individual patient 4Ts scoring parameters totaled to 7 are shown in Table 2.

**Table 2:** 4Ts for HIT score calculation in the patient HIT: Heparin-Induced Thrombocytopenia

4Ts	Clinical findings in the patient	Score
Thrombocytopenia	Platelet count fall 30%-50% OR platelet nadir 10-19	1
Timing of onset after heparin exposure	Clear onset between days 5-10 or platelet fall less than or equal to 1 day (prior heparin exposure within 30 days)	2
Thrombosis or other clinical sequelae	New thrombosis or skin necrosis: acute systemic reaction post-IV heparin bolus	2
Other causes of thrombocytopenia	None apparent	2

The heparin-induced platelet (HIP) antibody was ordered, which is a serologic screening test, as the next testing according to standards of care. The following day, the platelet counts further decreased to 77 K/μL, and the Heparin infusion was discontinued. Argatroban infusion was started, and Hematology was consulted. The hematologist evaluated the patient and confirmed that the clinical findings were consistent with HIT. He recommended continuing the argatroban infusion pending the results of the HIP antibody. By day 7, the Heparin-induced platelets antibody resulted positive, and the patient's optical density (O.D) was elevated to 0.536. The enzyme-linked immunosorbent assay (ELISA) HIP antibody test is ordered first because it serves as a screening test for patients with intermediate or high 4Ts scores for evaluating the likelihood of HIT and has a faster turnaround time, but a low specificity. The HIP antibody test result and interpretation are shown in Table 3.

**Table 3:** Heparin-induced platelet antibody ELISA test HIP: Heparin-Induced Platelet Antibody; O.D: Optical Density; ELISA: Enzyme-Linked Immunosorbent Assay

HIP Interpretation		
Related results	Reference range	Patient result
Patient O.D (HIP)	0.000-0.399	0.536
HIP interpretation	Negative	Positive

A serotonin release assay (SRA), which is the confirmatory test for HIT, was ordered. The platelet count continued to trend down to 58 K/μL while on Argatroban drip. On day 8, the Patient was noted to have right upper extremity swelling, and the VL duplex upper extremity right showed acute occlusive DVT involving the right subclavian, axillary, brachial, and internal jugular vein. With a platelet count of 54 K/μL, Hematology recommended increasing the Argatroban dose to the maximum of the therapeutic range, aiming for a PTT of 70-80, as the patient continued to experience thromboses. Platelet count trend during hospitalization is shown in Table 4.

**Table 4:** Platelet count trend Ref: Reference

Day of admission	Platelet count ref range and unit 163-337 K/μL
Day 1 morning	169
Day 1 evening	152
Day 3 morning	145
Day 3 evening	140
Day 4	118
Day 5	98
Day 6	77
Day 7	64
Day 8	58
Day 9	54
Day 10	68
Day 11	95
Day 12	103
Day 13	115

The platelet count began trending up on day 10 of admission to 68 K/μL, and on day 11, the UFH SRA resulted positive, and this confirmed the diagnosis of HIT. UFH low dose showed 88% and 100% release, while high dose showed 8%. A positive result for the SRA occurs only when there is a release of serotonin of 20% or greater at low doses of UFH, which can be either 0.1 IU/mL or 0.5 IU/mL. Additionally, specificity must be confirmed by demonstrating a reduction of at least 50% in serotonin release with the high dose of UFH (100 IU/mL). The SRA has a sensitivity ranging from 88% to 100% and a specificity ranging from 89% to 100% for diagnosing HIT.

On day 12 of admission, the patient was transitioned to Eliquis 5mg bid with a platelet count of 103 K/μL. By day 13 of admission, the patient's platelet trended up to 115 K/μL.

## Discussion

This discussion explores the mechanisms, diagnostic approach, clinical implications, and management strategies for HIT in a patient with PE and DVT. The paradox of thrombocytopenia and thrombosis defines the clinical challenge in managing HIT. In patients with PE, heparin is frequently administered as part of the acute management to prevent further thrombus formation, promote anticoagulation, and prevent complications such as recurrent embolism. However, the use of heparin in this context must be carefully monitored for the development of HIT, as it complicates treatment decisions and worsens the prognosis [6].

HIT can be classified into two pathophysiological types: HIT type I and HIT type II. We focus on HIT type II, an immune-mediated adverse reaction to heparin characterized by a significant decrease in platelet count (typically >50% reduction from baseline) and an increased risk of thrombosis. This condition can occur in patients exposed to unfractionated heparin (UFH) or low-molecular-weight heparin (LMWH), though UFH is more frequently associated with HIT [1,2].

A small percentage of patients may experience a temporary mild decrease in platelet count after one to four days of receiving heparin treatment. The platelet levels seldom drop below 100,000/μL, usually return to normal even with continued heparin use, and typically do not result in clinical complications. This non-immune-mediated HIT, caused by a direct interaction between heparin and circulating platelets, is classified as HIT type I. A more serious form of HIT usually appears about five to 10 days after starting heparin treatment, with an average of seven to eight days, but can develop more rapidly in patients who have had prior heparin exposure. This happens when the immune system reacts to heparin by producing immunoglobulin (Ig)G antibody that targets platelet factor 4 (PF4), a protein released from the alpha granules of the platelets (PF4/H) in the presence of heparin. This reaction can lead to increased platelet activity and dangerous blood clots. This more severe reaction is known as HIT type II [7]. Heparin platelet factor 4 features strong platelet activation linked to rapid thrombin production, potentially resulting in venous and/or arterial complications of thrombosis. Thrombocytopenia occurs due to the extensive activation of platelets followed by their removal by the mononuclear phagocyte system, with the cells being sensitized through PF4/heparin/IgG complexes. Thrombosis results from activating multiple cell types, including platelets that release procoagulant microparticles, endothelial cells, neutrophils, and particularly monocytes expressing tissue factors that lead to heightened hypercoagulability in patients [8].

The risk of HIT is reassuringly low, under 0.1% in various situations, such as when using low molecular weight heparin (LMWH) for medical treatments (not including cancer), in obstetrics (except cesarean sections), or for minor trauma. Patients administered a single bolus of UFH, particularly in endovascular examinations, generally exhibit a low risk of HIT. Nevertheless, this risk is elevated for individuals who have received heparin continuously for several days within the preceding three months. After one month of heparin treatment, the risk of HIT is very low in all patients, regardless of the type of heparin used (UFH or LMWH) and the dosage administered. There is a high risk of HIT, exceeding 1%, for most patients treated with UFH, regardless of whether they receive a preventive or therapeutic dose. This risk is particularly significant in patients undergoing orthopedic surgery or those recovering from cardiac surgery involving cardiopulmonary bypass. The risk is also higher during curative intravenous treatments in medical patients. The observation that the rate of seroconversion is markedly higher following treatment with UFH than LMWH highlights the critical role antigen structure plays in this disease [8,9].

The diagnosis of HIT should be considered in any patient who develops thrombocytopenia following heparin exposure, mainly when there is a significant reduction in platelets (greater than 50% of baseline) within 5 to 10 days after starting heparin. However, in patients who have received heparin in the past 30 days, HIT can develop sooner (within 24-48 hours). In this case, a patient with PE receiving heparin therapy presents with thrombocytopenia, which raises suspicion for HIT. The development of thrombocytopenia may be accompanied by new or worsening thrombotic events, such as deep vein thrombosis, stroke, or PE [10]. In addition to the drop in platelet count, other clinical features that may suggest HIT include skin necrosis at heparin injection sites or new-onset venous thromboembolism, often occurring in unusual sites like the upper extremities or other non-physiologic locations. Diagnosing HIT involves clinical suspicion alongside laboratory tests. The 4Ts scoring system is commonly employed to assess the pretest probability of HIT based on four criteria: Thrombocytopenia, Timing of platelet count drop, Thrombosis (or other clinical sequelae), and other causes of thrombocytopenia. A higher 4T score indicates a greater likelihood of HIT.

Laboratory confirmation of HIT typically includes an enzyme-linked immunosorbent assay (ELISA) to detect the presence of anti-PF4/heparin antibodies. A positive result should be confirmed with a functional assay, such as the SRA, which directly measures platelet activation in response to the heparin-PF4 complex [1,2,6,10].

Difficulties distinguishing HIT from several conditions with similar clinical features require sharp clinical judgment and thorough laboratory evaluation. Other causes of thrombocytopenia besides HIT include immune thrombocytopenic purpura (ITP), thrombotic thrombocytopenic purpura, drug-induced thrombocytopenia, and disseminated intravascular coagulation. The patient in our study had other tests to rule out different etiologies, and a respiratory-limited viral panel for COVID-19/respiratory syncytial virus/influenza was negative. Pulmonology was consulted for mediastinal adenopathy findings on the Chest CT for possible Endobronchial ultrasound (EBUS) bronchoscopy, but they recommended that, considering acute PE, the patient would be at very high risk for EBUS/bronchoscopy. The pulmonologist preferred to follow up with the patient on an outpatient basis to decide about further intervention. Furthermore, conditions such as deep vein thrombosis, PE, or arterial thrombosis (e.g., stroke or myocardial infarction) can occur alongside thrombocytopenia, making it essential to distinguish them from thrombotic complications associated with HIT [11]. Symptoms may appear days after the first exposure to heparin, complicating the establishment of a direct link between heparin administration and symptom onset. This delay adversely affects the sensitivity of the clinical scoring system employed [12].

The management of HIT in a patient with PE involves immediate cessation of heparin and transition to an alternative non-heparin anticoagulant, such as direct thrombin inhibitors (e.g., argatroban or bivalirudin) or factor Xa inhibitors (e.g., fondaparinux), or direct oral anticoagulants (DOACs) (e.g., apixaban or rivaroxaban). Argatroban is a direct thrombin inhibitor given through a continuous intravenous infusion, and it has a brief duration of action, making it particularly suitable for patients who might need further surgical interventions or those at a high risk of bleeding. Importantly, the use of argatroban for HIT has faced criticism because it necessitates PTT monitoring, which often occurs in a critical care environment where an existing coagulopathy can interfere with PTT results, potentially leading to underdosing. Argatroban undergoes metabolism in the liver, which positions it as a suitable alternative for patients with renal impairment [6]. DOACs like apixaban, dabigatran, rivaroxaban, streamline the initial treatment, prevention, and ongoing management of PE since they can be given in set doses without requiring laboratory monitoring of coagulation [13]. Warfarin, though effective in managing PE, should not be used in the acute phase of HIT until platelet counts have recovered due to the risk of inducing a transient hypercoagulable state [14,15]. In addition to anticoagulation, careful monitoring of platelet counts and assessment for thrombotic events is essential. The decision to continue for three to six months or discontinue long-term anticoagulation will depend on the patient's overall clinical status, risk of recurrence, and whether the PE was provoked or unprovoked [5,6]. A patient with an HIT diagnosis must have this noted in their clinical history to avoid future exposure to heparin within 100 days because the IgG remains for 100 days. Beyond that, use is controversial but may be attempted in low doses. Ongoing patient education about HIT, the need for alternative anticoagulants, and the awareness of thrombotic complication signs are essential [16].

Prognosis

The outcome of HIT primarily hinges on prompt detection and suitable treatment. By swiftly stopping heparin and initiating appropriate alternative anticoagulation therapy, the mortality linked to HIT has notably diminished. Conversely, if not treated or if there are delays in management, HIT can result in serious complications, such as limb gangrene, stroke, and death from thrombotic incidents. Untreated acute PE is associated with high mortality rates. Early heparin administration is crucial for survival, and anticoagulation has been the cornerstone of PE treatment for decades. The primary goal of treatment is to reduce mortality by preventing the extension of existing blood clots, the formation of new thrombi, and further embolization.

## Conclusions

HIT in those with PE represents a notable diagnostic and treatment challenge. This issue highlights the need for vigilant monitoring of platelet levels during heparin therapy, particularly for patients suspected of or confirmed to have venous thromboembolism. Early detection of HIT, along with the swift cessation of heparin and the start of alternative anticoagulants, is crucial to lessen morbidity and mortality in this vulnerable group. Timely intervention and a multidisciplinary approach to anticoagulation management are critical for optimizing patient outcomes.
